# Type IV secretion systems: tools of bacterial horizontal gene transfer and virulence

**DOI:** 10.1111/j.1462-5822.2008.01187.x

**Published:** 2008-07-08

**Authors:** Mario Juhas, Derrick W Crook, Derek W Hood

**Affiliations:** 1 Clinical Microbiology and Infectious Diseases, Nuffield Department of Clinical Laboratory Sciences, University of OxfordOxford OX3 9DU, UK; 2 Molecular Infectious Diseases Group, The Weatherall Institute of Molecular Medicine, University of OxfordOxford OX3 9DS, UK

## Abstract

Type IV secretion systems (T4SSs) are multisubunit cell-envelope-spanning structures, ancestrally related to bacterial conjugation machines, which transfer proteins and nucleoprotein complexes across membranes. T4SSs mediate horizontal gene transfer, thus contributing to genome plasticity and the evolution of pathogens through dissemination of antibiotic resistance and virulence genes. Moreover, T4SSs are also used for the delivery of bacterial effector proteins across the bacterial membrane and the plasmatic membrane of eukaryotic host cell, thus contributing directly to pathogenicity. T4SSs are usually encoded by multiple genes organized into a single functional unit. Based on a number of features, the organization of genetic determinants, shared homologies and evolutionary relationships, T4SSs have been divided into several groups. Type F and P (type IVA) T4SSs resembling the archetypal VirB/VirD4 system of *Agrobacterium tumefaciens* are considered to be the paradigm of type IV secretion, while type I (type IVB) T4SSs are found in intracellular bacterial pathogens, *Legionella pneumophila* and *Coxiella burnetii*. Several novel T4SSs have been identified recently and their functions await investigation. The most recently described GI type T4SSs play a key role in the horizontal transfer of a wide variety of genomic islands derived from a broad spectrum of bacterial strains.

## Introduction

Many bacterial species exploit specialized secretion systems to transfer macromolecules across membranes. These secretion systems are assembled into six major groups, named types I, II, III, IV, V and VI (Thanassi and Hultgren, 2000; Henderson *et al*., 2004; Mougous *et al*., 2006). The secretion systems ancestrally related to the bacterial conjugation machinery are referred to as the type IV secretion systems (T4SSs) (Lawley *et al*., 2003; Christie *et al*., 2005). The T4SSs are unique among other bacterial secretion system types due to their ability to transfer both proteins and nucleoprotein complexes.

**Fig. 1:**
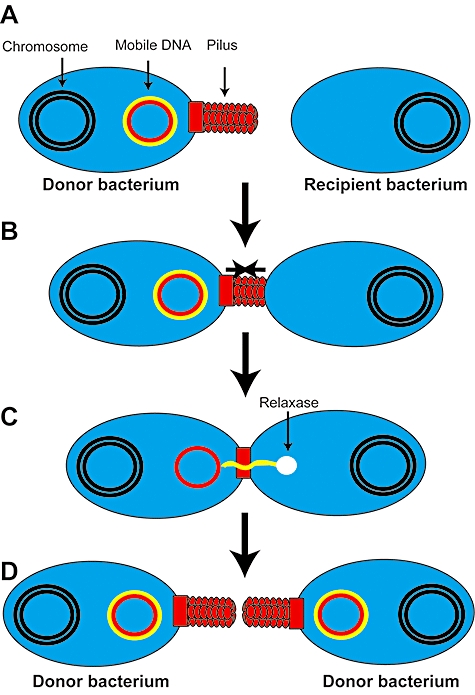
Conjugation. Conjugation systems (shown is the shot-and-pump model of conjugative DNA transfer) represent a large subfamily of the T4SSs and are used by bacteria in the process of the conjugative transfer of DNA from donor to recipient cells (A) by cell-to-cell contact usually mediated by the retraction of the pilus-like structures (B). C. ssDNA of the mobile genetic element is transferred from the donor to recipient bacteria with the help of the relaxase. D. Complementary DNA strands are synthesized in both cells and the former recipient bacterium becomes a new potential donor of the mobile DNA.

T4SSs are multisubunit cell-envelope-spanning structures comprising a secretion channel and often a pilus or other surface filament or protein (Lawley *et al*., 2003; Christie *et al*., 2005). Research on T4SSs began with the discovery of the F plasmid-borne conjugation system few decades ago. Conjugation systems, including the one encoded by the F plasmid, represent a large subfamily of the T4SSs and are used by bacteria in the process of the conjugative transfer of DNA from donor to recipient cells (Fig. 1). By conjugation, T4SSs mediate horizontal gene transfer, thus contributing to genome plasticity and the evolution of infectious pathogens through dissemination of antibiotic resistance and virulence genes. The T4SS of *Neisseria gonorrhoeae*, evolutionarily related to the conjugation machinery, mediates secretion of naked DNA into the extracellular environment instead of the donor cells (Hamilton *et al*., 2005). Moreover, T4SSs are also used for the delivery of bacterial effector proteins across the bacterial membrane and the plasma membrane of eukaryotic target cells, thus contributing directly to the bacterial pathogenicity (Christie *et al*., 2005).

## Type IV secretion systems: a challenge to simple classification

T4SSs are encoded by multiple genes organized into a single functional unit. The T4SSs gene clusters hitherto described differ significantly in a number of respects, including gene content, gene order and the number of homologues they share. Based on the number of features, including the organization of genetic determinants, shared homologies and evolutionary relationships, T4SSs have been classified into major types using two different classification schemes (Lawley *et al*., 2003; Christie *et al*., 2005).

In the original classification scheme there were initially three major types, referred to as types F, P and I, based on the incompatibility group of the representative conjugative plasmids, IncF (plasmid F), IncP (plasmid RP4) and IncI (plasmid R64) respectively (Lawley *et al*., 2003). In the alternative classification schemes, types F and P have been grouped together as type IVA systems, which resemble the archetypal VirB/VirD4 system of *Agrobacterium tumefaciens*. Type I, which varies significantly in its component modules from members of both F and P types, was named as the type IVB system. Genetic determinants of the type IVB systems are related to the archetypal Dot/Icm system of *Legionella pneumophila.* A third group in this classification scheme, composed of all the ‘other’ T4SS representatives that bear no or only limited homology to IVA and IVB system, has not been well characterized (Christie *et al*., 2005). Representatives of this group include for instance the recently identified GI lineage of T4SSs associated with broad spectrum of genomic islands in various bacteria (see below) (Juhas *et al*., 2007a).

In both classification schemes, members within each type exhibit evidence of common ancestry. Within each type there is greater conservation of gene content and order than there is between types. Alignment of amino acid sequences shared by homologous proteins supports the division into these groups.

More recently, a novel lineage of T4SSs has been identified on the genomic island ICE*Hin1056*, which is a vector of antibiotic resistance in *Haemophilus influenzae*. This T4SS is distinct from all previously described types. It represents a fourth lineage with a genetic distance as great as is observed between the F, P and I lineages. Using the alternative classification system (Christie *et al*., 2005), this new T4SS would be classified as ‘other’. This novel type of T4SSs is present in a wide variety of related syntenic genomic islands including pKLC102, PAPI, SPI-7 and the *clc* element as well as others derived from a broad variety of bacterial strains including *Pseudomonas aeruginosa, Pseudomonas fluorescens, Erwinia carotovora, Salmonella enterica* serovar Typhi and *L. pneumophila.* This lineage of T4SSs has been named GI type in order to emphasize the fact that this T4SS is found so far associated only with genomic islands (Juhas *et al*., 2007a).

Relationships within and among T4SS types are based mainly on three genes, named *traB*/*virB10, traC*/*virB4* and *traD*/*virD4* (named after genes on the *Escherichia coli* F plasmid or on the *A. tumefaciens* Ti plasmid respectively). As shown in our study, the high sequence variability of *traD* means that it is not an ideal candidate for phylogenetic analysis, whereas genes *traB*/*virB10* and *traC*/*virB4* encode quintessential T4SS proteins of Gram-negative bacteria that are ideal for comparative amino acid alignment and as such appear sufficient to define membership of a T4SS type (Juhas *et al*., 2007a). If these two genes provide the key signature that could identify other more distantly related T4SSs in genomes and, in particular, genomic islands, searching for homologues of these genes might be a way to identify other distinct T4SS lineages. However, it should be noted that, as *traB*/*virB10* is not a component of conjugation systems of Gram-positive bacteria, *traC*/*virB4* remains the only universally conserved T4SS component of both Gram-negative and Gram-positive bacteria.

The division of T4SSs into four groups: F, P, I, and GI seems to provide a sound framework for classifying the majority of T4SSs. It is only recently that the increased availability of genome sequence data and improved bioinformatic techniques have allowed the recognition of the novel GI type of T4SS. Thus, it is quite realistic to assume that with the use of modern computational biology techniques, other new divergent T4SS types will be identified in the forthcoming years.

## F and P type IV secretion systems: paradigm of type IV secretion

**Fig. 2:**
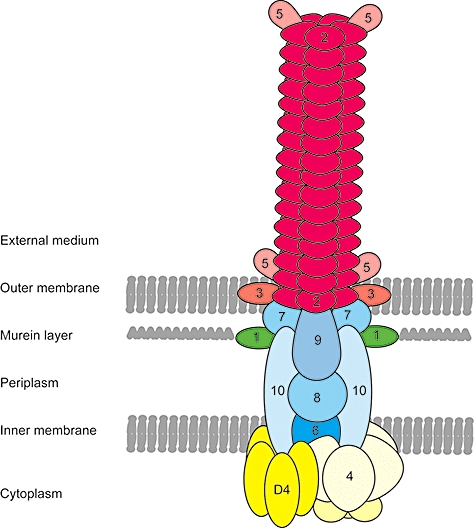
Model of the VirB/VirD4 type IV secretion machinery of *Agrobacterium tumefaciens.* The T4SS of *A. tumefaciens* is a multicomponent cell-envelope spanning structure that consists of 11 VirB proteins VirB1–VirB11 and VirD4. Colour code: yellow, nucleoside triphosphatases that provide energy for the transfer; blue, components of the transmembrane channel; red, pilus-forming components; green, lytic transglycosylase responsible for the degradation of the murein (peptidoglycan) layer at the site of assembly.

The F and P type T4SSs, also referred to as type IVA systems, resemble the archetypal VirB/VirD4 system of *A. tumefaciens*. The T4SS of *A. tumefaciens* is encoded by an approximately 10 kb ‘*virB* operon’, comprising 11 open reading frames and a separate *virD4* gene, and mediates transfer of oncogenic genes into plant cells, resulting in tumorigenesis and subsequent crown gall disease. Most of the information concerning functional properties of T4SSs has been obtained from the study of the VirB/VirD4 system of *A. tumefaciens*. According to the most recent views, of the 11 VirB protein determinants of the *A. tumefaciens* VirB/VirD4 T4SS, proteins VirB2 and VirB5 are pilus components, VirB3 and VirB7 are pilus-associated proteins, VirB4 and VirB11 are nucleoside triphosphatases that provide energy for transfer, while VirB6, VirB7, VirB8, VirB9 and VirB10 constitute components of the transmembrane channel (Fig. 2). VirB1 is a lytic transglycosylase that degrades the peptidoglycan cell wall at the site of T4SS assembly, and VirD4 is another nucleoside triphosphatase, called ‘coupling protein’, which recruits DNA to the components of the secretion system (Christie *et al*., 2005; Backert and Meyer, 2006; Fig. 2). Interestingly, the C-terminal part of VirB1, designated VirB1* was shown to be cleaved and secreted from cells where it was involved in the formation of the T4SS pilus (Zupan *et al*., 2007). Thus, VirB1 acts as a bifunctional protein that lyses peptidoglycan cell wall to facilitate insertion of the T4SS but simultaneously also promotes formation of the pilus through interaction with pilus subunits (Zupan *et al*., 2007). The recruitment of the transfer substrate of *A. tumefaciens* VirB/VirD4 T4SS consisting of T-DNA encoding oncogenic proteins and VirD2 relaxase was shown to be facilitated by the previously unrecognized recruiting protein VBP (VirD2-binding protein; Guo *et al*., 2007). In addition to the T-DNA-encoding oncogenic proteins bound to the VirD2 relaxase, the VirB/VirD4 T4SS of *A. tumefaciens* transfers several effector proteins into the host plant cells: namely VirD5, VirE2, VirE3 and VirF that increase the chance of successful infection (Vergunst *et al*., 2000). Recent work showed that VirE2 acts as a unique powerful ssDNA-binding molecular machine that mediates infection by actively pulling T-DNA into the host cells without the need for external energy sources (Grange *et al*., 2008).

Several other T4SSs sharing a common ancestry with the VirB/VirD4 system of *A. tumefaciens* have been identified. Some of them contain the complete set of *A. tumefaciens virB*/*virD4* genes, while others are chimeras of the *virB*/*virD4* and other unrelated genes (Christie *et al*., 2005). One good example is from *Helicobacter pylori*, the causative agent of gastritis, peptic ulcer and gastric cancer in humans (Blaser and Atherton, 2004). Most virulent strains of *H. pylori* harbour the *cag* pathogenicity island encoding a VirB/VirD4-like T4SS. T4SS of *H. pylori* contains genes encoding proteins homologous to VirB4, VirB7, VirB9, VirB10, VirB11 and VirD4 of *A. tumefaciens*, in addition to other genes with unknown function. Furthermore, topological and mutational analyses suggest similar functions to *A. tumefaciens* proteins VirB1, VirB2, VirB6 and VirB8 for four additional non-homologous *H. pylori* proteins (Buhrdorf *et al*., 2003; Andrzejewska *et al*., 2006). Most recent study aiming at the elucidation of the structure of the *H. pylori* T4SS apparatus has shown that *H. pylori* T4SS contains functional analogues of all components of the VirB/VirD4 system of *A. tumefaciens*, except VirB5 (Kutter *et al*., 2008). The only effector protein of the *H. pylori* T4SS known to date, CagA, interacts with several host cell proteins, resulting in altered physiology of the host cells and an increased chance of successful infection (Bagnoli *et al*., 2005; Bourzac and Guillemin, 2005; Brandt *et al*., 2005; Bourzac *et al*., 2007; Moese *et al*., 2007; Zeaiter *et al*., 2008). The exact mechanism by which CagA is translocated by *H. pylori* T4SS remained elusive for a long time; however, a recent study involving fusion proteins and immunoprecipitation studies led to identification of the CagF protein, that is involved in the interaction with CagA (Couturier *et al*., 2006). Results from this study indicate that CagF is a protein that recognizes and delivers CagA into the T4SS channel (Couturier *et al*., 2006). Another protein crucial for the successful delivery of CagA into host cells is the specialized adhesin CagL. Located on the surface of the T4SS pilus, CagL triggers delivery of CagA via activation of integrin receptors on the surface of the host cells (Kwok *et al*., 2007).

A VirB/VirD4-like T4SS plays an important role in the pathogenesis of the intracellular *Bartonella* spp. (Schmid *et al*., 2004). Several translocated effector proteins (Beps) are involved in a wide variety of virulence associated traits, including activation of pro-inflammation, apoptosis and cytoskeleton rearrangements (Schulein *et al*., 2005; Backert and Meyer, 2006). A related T4SS is also exploited by the causative agent of the whooping cough, *Bordetella pertussis* to deliver pertussis toxin into the extracellular milieu (Rambow-Larsen and Weiss, 2004). Recently, presented model suggests that during first stage pertussis toxin interacts in the periplasm with the partially assembled secretion apparatus and only after this initial interaction, the complete T4SS is assembled and secretion of the pertussis toxin across the outer membrane can proceed (Verma and Burns, 2007).

## I type IV secretion systems: protein secretion machines of intracellular pathogens

The I type T4SSs, also referred to as type IVB systems, resembling the archetypal Dot/Icm system of the IncI plasmids have been identified in two intracellular bacterial pathogens, *L. pneumophila* and *Coxiella burnetii*.

*Legionella pneumophila* is a Gram-negative facultative intracellular parasite found primarily associated with environmental amoebae and protozoa, but it is better known as the causative agent of a community acquired or nosocomial pneumonia called Legionnaires' disease. After successful infection through contaminated aerosols, *L. pneumophila* is phagocytosed by alveolar macrophages that do not undergo subsequent lysis due to the inhibition of the phagosome–lysosome fusion at early stages of phagosome maturation (Wiater *et al*., 1998). Twenty-five open reading frames whose products are crucial for the intracellular survival of the bacterium in macrophages have been identified and named *icm* (intracellular multiplication) and *dot* (defective organelle trafficking) genes. A significant number of the Dot/Icm proteins are homologous to the components of the conjugation system of the IncI plasmids such as R64 (Vogel *et al*., 1998; Komano *et al*., 2000). The Dot/Icm T4SS of *L. pneumophila* is absolutely required for virulence of this bacterium, as T4SS mutants are impaired in a variety of pathogenic properties. These include phagocytosis, pore formation in the host cell membranes and inhibition of phagosome–lysosome fusion. Furthermore, the Dot/Icm T4SS of *L. pneumophila* is involved in the recruitment of the rough endoplasmic reticulum that, together with phagosomes, forms a favourable niche for the replication of the bacterium inside the host macrophage. This T4SS also promotes apoptosis of the host cells and escape of the bacterium from the phagosome (Segal *et al*., 2005; Robinson and Roy, 2006). Several effector proteins secreted by the Dot/Icm secretion systems have been identified. One of them is RalF, a protein containing a Sec-7 homology domain typical of eukaryotic ARFs (ADP ribosylation factors), which is presumably used for the activation of ARF through exchange of GDP for GTP and subsequent ARF-mediated recruitment of endoplasmic reticulum vesicles (Nagai *et al*., 2002). Other proteins include LidA, required for the formation of the replicative vacuole, LepA and LepB, crucial for the escape of bacteria from the phagosome and numerous Sids (substrates of Icm/Dot transporter), whose function in *L. pneumophila* virulence is currently under intensive investigation (Conover *et al*., 2003; Luo and Isberg, 2004; Segal *et al*., 2005). SidM (DrrA) is a bifunctional enzyme involved in the activation of Rab1 through exchange of GDP to GTP and in the recruitment of Rab1 to the *Legionella-*containing vacuole (Ingmundson *et al*., 2007; Machner and Isberg, 2007). SidJ is required for efficient recruitment of endoplasmic reticulum to the bacterial phagosome (Liu and Luo, 2007) and SidF inhibits host cells from undergoing apoptosis to achieve maximal bacterial multiplication (Banga *et al*., 2007). Components of the Dot/Icm T4SS of *L. pneumophila*, together with numerous translocated effector proteins of this system were shown to be under the direct control of two regulators, PmrA and CpxR (Zusman *et al*., 2007; Altman and Segal, 2008). A comprehensive biochemical and genetic examination of the *L. pneumophila* T4SS was reported that shed more light into its structure (Vincent *et al*., 2006). Dot/Icm subcomplex consisting of five proteins, DotC, DotD, DotF, DotG and DotH, was identified that represents the core of the secretion complex and bridges the inner and outer membranes of *L. pneumophila*. This subcomplex seems to be functionally analogous to the *A. tumefaciens* VirB7–VirB10 subcomplex, thus suggesting a remarkable conservation of the core structure in these evolutionary distant T4SSs (Vincent *et al*., 2006).

*Coxiella burnetii* is a Gram-negative obligate intracellular bacterial pathogen of animals and the causative agent of Q fever in humans. After phagocytosis by host alveolar macrophages, *C. burnetii* delays phagosome–lysosome fusion, presumably to change from small-cell variants to large-cell variants, a process that allows this bacterium to thrive in the acidic environment of the phagolysosome (Zamboni *et al*., 2003; Parker *et al*., 2006). Initial sequence analysis of the *C. burnetii* genome revealed that it contains a majority of the *L. pneumophila* T4SS *dot*/*icm* genes, with the exception of *icmR, dotJ* and *dotV*; however, a protein non-homologous but functionally similar to IcmR of *L. pneumophila* was identified in the later study (Feldman *et al*., 2005). *icm*/*dot* genes of *C. burnetii* are clustered in a single region on the chromosome in contrast to *L. pneumophila* where these genes are present in two separate locations (Sexton and Vogel, 2002; Segal *et al*., 2005). Similarly to *L. pneumophila*, T4SS of *C. burnetii* was shown to be under the direct control of the regulatory protein PmrA (Zusman *et al*., 2007). Several of the *C. burnetii icm*/*dot* genes can substitute their homologues in *L. pneumophila,* but their direct involvement in *C. burnetii* virulence has not been investigated.

## GI type IV secretion systems and their role in horizontal gene transfer

The horizontal gene pool contributes to the diversification and adaptation of microorganisms, thus having a significant impact on the genome plasticity of environmental bacterial species, the evolution of bacterial pathogens, and dissemination of antibiotic resistance genes and other virulence factors (Christie *et al*., 2005).

A major part of the horizontal gene pool consists of genomic islands which are mobile segments of bacterial genomes often inserted at tRNA genes and flanked by direct repeat sequences and whose G+C content differs from the rest of the chromosome. They often contain homologues of integrases and transposases and other genes associated with conjugative plasmids or phages. Furthermore, genomic islands often harbour a variable number of genes offering a selective advantage for host bacteria, such as metabolic, antibiotic resistance or virulence genes. According to their gene content, genomic islands are often described as pathogenicity, symbiosis, metabolic, fitness or resistance islands (Dobrindt *et al*., 2004).

Identification of a novel GI-like group of T4SSs brought new insight into the mechanism by which genomic islands transfer between bacteria. It was generally thought that genomic islands represent mobile elements such as conjugative plasmids that have co-integrated with the chromosome and lost their ability to further self-transfer (Dobrindt *et al*., 2004). This hypothesis has been challenged by experiments performed with the representatives of a family of syntenic genomic islands with deep evolutionary origin that includes ICE*Hin1056* of *H. influenzae* and the *clc* element, pKLC102 and PAPI of *Pseudomonas* spp. Several representatives of this family of genomic islands can transfer between bacterial cells after previous integration into the chromosome (Dimopoulou *et al*., 2002; van der Meer and Sentchilo, 2003; Klockgether *et al*., 2004; Mohd-Zain *et al*., 2004). A combination of bioinformatics and functional analyses has revealed a highly conserved set of genes encoding a novel GI-like lineage of T4SS that plays a key role in the horizontal transfer of these genomic islands (Juhas *et al*., 2007a).

First functional analysis of the GI-like T4SSs has been performed with the genomic island ICE*Hin1056* from *H. influenzae*, which confers resistance to ampicillin, chloramphenicol and tetracycline. Prior to 1972, *H. influenzae* was universally susceptible to ampicillin. In 1972, the first ampicillin-resistant isolate was detected and soon afterwards tetracycline, chloramphenicol, erythromycin and multiple antibiotics-resistant strains were identified that spread rapidly around the globe. Recent work has shown that a novel GI-like lineage of T4SSs plays a key role in the dissemination of the antibiotic resistance element ICE*Hin1056* among *Haemophilus* spp. Several genes of the GI T4SS of *H. influenzae* harboured transmembrane domains and signal peptide sequences, features typical for genes involved in T4SS (Table 1). Mutation analysis showed that inactivation of key genes of the ICE*Hin1056* T4SS resulted in a loss of phenotypic traits provided by a T4SS. Several mutants with a mutation in this T4SS did not produce the type IV secretion pilus and had up to 100 000-fold reduced conjugation frequencies compared with the parent strain (Juhas *et al*., 2007a; Table 1). In a subsequent study investigating the sequence and functional properties of various *Haemophilus* spp. genomic islands, the GI-like T4SS module was found to be among the most conserved parts of seven genomic islands tested, with DNA similarity ranging from 95% to 100% between islands. Furthermore, results from this study suggest that GI T4SSs of all *Haemophilus* genomic islands tested play a key role in the formation of the pilus and conjugative transfer of DNA (Juhas *et al*., 2007b).

**Table 1:** The GI T4SSs.

	Gene name (*tfc*)																							
	1	2	3	4	5	6	7	8	9	10	11	12	13	14	15	16	17	18	19	20	21	22	23	24
Transmembrane domains	+	−	−	−	−	−	−	+	−	+	+	−	−	−	−	−	−	+	+	+	−	−	−	−
Signal peptide sequence	−	+	+	+	+	−	−	−	+	−	−	+	−	+	+	−	−	−	−	−	+	+	−	−
Pilus formation	−	+	un	+	un	−	un	un	un	un	un	+	un	+	un	+	un	un	un	un	un	+	+	−
Conjugation	+	+	un	+	un	+	un	un	un	un	un	+	un	+	un	+	un	un	un	un	un	+	+	+

Table shows characteristics (transmembrane domains, signal peptide sequences, role in the pilus formation and conjugation) of the individual gene components of the GI T4SSs. un = unknown.

This novel group of T4SSs is also harboured by a broad spectrum of other genomic islands with different properties, ranging from virulence and antibiotic resistance to biodegradation. Besides pKLC102 and PAPI of *P. aeruginosa* (Klockgether *et al*., 2007; Wurdemann and Tummler, 2007), examples include the *clc* element from *Pseudomonas* sp. strain B13 carrying the genetic information for several degradation pathways, including chlorobenzoate and chlorocatechol degradation (van der Meer and Sentchilo, 2003; Gaillard *et al*., 2006), or SPI-7 of *S. enterica* serovar Typhi encoding Vi polysaccharide antigen and SopE effector protein of the SPI-1 system (Baker *et al*., 2008). Furthermore, GI-like T4SSs are also harboured by genomic islands of an anaerobic aromatic-degrading denitrifying bacterium *Azoarcus* sp. strain EbN1 (Rabus *et al*., 2005), plant pathogen *E. carotovora* ssp. atroseptica SCRI1043 (Chen *et al*., 2008), methylotrophic bacterium *Methylibium petroleiphilum* PM1 (Kane *et al*., 2007), insect pathogen *Photorhabdus luminescens* TT01 (Held *et al*., 2007), plant pathogen *Xylella fastidiosa* (Zaini *et al*., 2008), human enteropathogen *Yersinia enterocolitica* strain 8081 (Thomson *et al*., 2006), *Yersinia pseudotuberculosis* 32777 (Collyn *et al*., 2006) and numerous sequenced *Pseudomonas* strains (Klockgether *et al*., 2007).

**Fig. 3:**
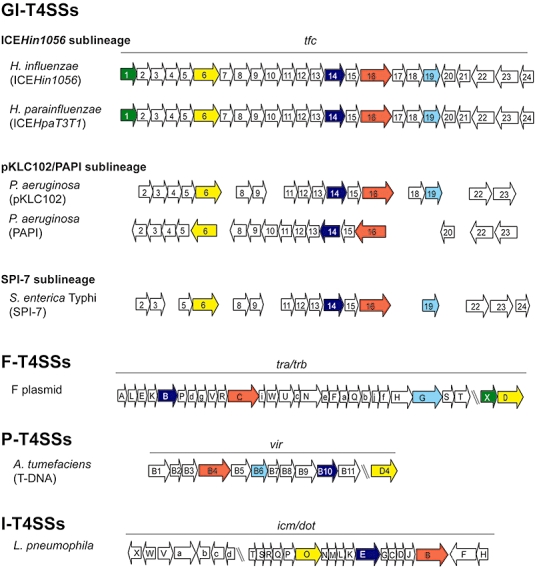
The variable T4SSs. The picture shows genetic organization of the GI T4SS in three known sublineages (ICE*Hin1056*, pKLC102/PAPI, SPI-7) and its homologues in paradigmal F and P-like T4SSs and I-like T4SSs. GI T4SSs are well conserved in genomic islands from different bacterial species and share only limited homology to few components of the F- and P- and I-like T4SSs. Genes homologous across T4SS groups are highlighted with the same colour. Upper case gene names = Tra, Icm and lower case gene names = Trb, Dot for F plasmid and *L. pneumophila* respectively.

Recent findings indicate that GI-like T4SSs can be further divided into three major sublineages (Klockgether *et al*., 2007; Fig. 3). First, ICE*Hin1056* sublineage, includes T4SSs of genomic islands found in variety of *Haemophilus* spp. (Juhas *et al*., 2007b), while second, pKLC102/PAPI sublineage comprises T4SSs of metabolically very versatile group of bacteria, including different *Pseudomonas* spp., *Azoarcus*, *M. petroleiphilum* and *X. fastidiosa* (Klockgether *et al*., 2007). Third, SPI-7 sublineage, comprises T4SSs of genomic islands of the enteropathogen *S. enterica* serovar Typhi, *E. carotovora* and *P. luminescens*. GI-like T4SSs are highly conserved between genomic islands of all three sublineages (Fig. 3). The complete set of 24 genes has been identified in all *H. influenzae* and *Haemophilus parainfluenzae* genomic islands tested so far. Furthermore, almost the whole sets of the GI-like T4SSs genes have been also identified in genomic islands of the pKLC102/PAPI and SPI-7 sublineages (Fig. 3). On the other hand, only a few evolutionary distant homologues of the GI T4SS have been found in the previously described paradigmal F- and P-like T4SSs and I-like T4SSs of intracellular pathogens (Fig. 3).

However, despite strong conservation of this T4SS, it should be noted that there are significant differences in mobility and expression of GI T4SS-harbouring genomic islands. For example, T4SS-dependent conjugative transfer of ICE*Hin1056* between two *H. influenzae* strains proceeds at various frequencies ranging from 10^−1^ to 10^−9^ (Juhas *et al*., 2007b). The *clc* element of *Pseudomonas* sp. strain B13 is transferable at frequencies of around 10^−1^−10^−2^ to *Pseudomonas putida* and *P. aeruginosa* (Gaillard *et al*., 2006; Gaillard *et al*., 2008). pKLC102 and PAPI-1 of *P. aeruginosa* are capable of self-transfer at frequencies similar to the *clc* element, but no conjugative transfer has been demonstrated for other members of this sublineage, like PAGI-2 and PAGI-3 (Qiu *et al*., 2006; Klockgether *et al*., 2007). Similarly, no conjugative transfer has been shown for the SPI-7 of *S. enterica* serovar Typhi (Baker *et al*., 2008). Variations in the conjugation frequencies of the more distant genomic islands could be attributed to the slight differences in the gene content of their T4SS modules; however, this explanation would not suffice for the closely related islands with the same set of 24 GI T4SS genes like ICE*Hin1056* and ICE*HpaT3T1* (Fig. 3). Rather, this could be related to the differences in the host strain. Recent findings indicate that host strain background has a tremendous impact on the conjugal transfer efficiency and expression of genomic islands (Juhas *et al*., 2007b; Gaillard *et al*., 2008).

In conclusion, GI-like T4SSs allow genomic islands to mobilize and spread through a bacterial population, thus playing a key role in bacterial virulence, evolution and adaptation to variable environments. What remains to be seen is whether the many and diverse genomic islands not related to ICE*Hin1056*, pKLC102/PAPI and SPI-7 have a similar secretion machinery that enables them to propagate rapidly through horizontal gene transfer.

## Conclusions

Due to the contribution of a number of researchers worldwide, type IV secretion is one of the most rapidly advancing fields of research in microbiology. T4SSs mediate the horizontal transfer of genes, thus contributing to the plasticity of bacterial genomes and transmission of antibiotic resistance genes and other fitness factors between bacterial species. Furthermore, T4SSs are used for the delivery of bacterial effector proteins into the eukaryotic host cells. Since the discovery of the transfer region of the conjugative F plasmid and Ti plasmid of *A. tumefaciens*, similar systems have been discovered in a wide variety of other bacteria and it is reasonable to predict that many more will be discovered with the increasing number of bacterial genomes being sequenced. Functional studies and detailed characterization of the components of T4SSs diverse from the archetypal T4SSs will enhance our understanding of the function and diversity of bacterial secretion machines.
